# Arabinogalactan Proteins Mark the Generative Cell–Vegetative Cell Interface in Monocotyledonous Pollen Grains

**DOI:** 10.3390/cells14191549

**Published:** 2025-10-03

**Authors:** Małgorzata Kapusta, Magdalena Narajczyk, Bartosz J. Płachno

**Affiliations:** 1 Bioimaging Laboratory, Faculty of Biology, University of Gdańsk, 59 Wita Stwosza St., 80-308 Gdansk, Poland; 2 Department of Plant Cytology and Embryology, Institute of Botany, Faculty of Biology, Jagiellonian University in Kraków, 9 Gronostajowa St., 30-387 Krakow, Poland

**Keywords:** arabinogalactan proteins, immunolocalization, monoclonal antibodies, monocotyledons, gametic cells, generative cell, pollen grains

## Abstract

Arabinogalactan proteins (AGPs: hydroxyproline-rich glycoproteins) are ubiquitous in plants and play various functions in cases of development and reproduction. In *Arabidopsis thaliana* some AGPs can work as markers for gametophytic cell differentiation (among others embryological structures they mark generative cell wall and/or plasma membrane, and also sperm cells). However, apart from *Arabidopsis*, this labeling of generative cell and sperm cells in pollen grains has only been observed in a few flowering plant species belonging to dicotyledons. No such studies are available in monocotyledons. The main aim of our study was to see whether AGPs would be present at the generative cell–vegetative cell interface in different monocotyledons (representatives of Asparagaceae, Amarylidaceae and Liliaceae), and we also wanted to test whether they would be the same AGPs as in dicotyledons. For the study, we selected *Gagea lutea* (L.) Ker Gawl., *Ornithogalum nutans* L. and *Galanthus nivalis* L. species that differ in shape and size of generative cells. Antibodies against arabinogalactan proteins AGPs were used, including JIM8, JIM13, JIM14, MAC207, LM2, LM14, JIM15 and JIM4. The localization of the examined compounds was determined using immunohistochemistry techniques. The key finding was that AGPs (detected with JIM8 and JIM13 antibodies) consistently mark the boundary between the generative cell and the surrounding vegetative cytoplasm, suggesting their association with the generative cell–vegetative cell interface in all species studied. Identifying such molecular markers in male gametophyte may enhance the understanding of gametophytic cell fate, sperm cell identity and the molecular mechanisms underlying fertilization. Such labeling may also be useful in studies on pollen development, species comparisons, or responses to environmental stresses.

## 1. Introduction

Pollen, which carries male gametes, plays a crucial role in flowering plants as a partner for ovules in double fertilization [1,2,3]. The male gametophyte develops from a microspore, which undergoes vacuolization and then asymmetrically divides into a generative cell (GC) and a vegetative cell (VC) [4,5,6,7,8,9,10,11].

Some monocotyledonous plants produce bicellular mature pollen with pollen mitosis II (PMII) occurring within the pollen tube. The detachment of the generative cell from the sporoderm to the interior of the vegetative cell appears to be a unique cellular process specific to flowering plants. The GC remains submerged in the cytoplasm of the vegetative cell throughout its life [10,12]. The generative cell may divide within the pollen and then the mature pollen is trinucleate, or only inside the pollen tube and the mature pollen is then binucleate [13]. Following the division of the generative cell, the male gametophyte can be considered fully mature [14,15].

Proper formation of the pollen wall is essential for maintaining cell integrity during male germ differentiation, and disturbances in this process may lead to defective pollen grains and failure of germination [15]. The generative cell and, later, the two sperm cells are enclosed by a plant-derived membrane termed the peri-germ cell membrane (PGCM), which separates them from the surrounding vegetative cytoplasm [16]. In this study, we refer to this region as the generative cell–vegetative cell interface, acknowledging that light microscopy cannot unambiguously distinguish between the PGCM, the generative cell plasma membrane, or the intermembrane apoplastic space [16].The cell wall is a unique structure, not only providing cells with rigidity but also actively participating in fundamental developmental processes such as reproduction or stress responses [17,18]. In primary cell walls, the matrix embedding the cellulose network is composed of pectin—a highly hydrated network of polysaccharides rich in galacturonic acid [19]. Cell wall architecture can vary not only between organs but also during the life of a single cell. Remodeling of the cell wall plays a pivotal role in determining cell fate. Specialization of the cell wall may involve certain regions, known as microdomains, which differ in composition from the surrounding wall and play critical roles in cellular functions [20]. These microdomains can influence the function of plant organs by regulating processes such as growth and stiffness. For example, the incorporation of hemicelluloses into cell wall components is critical for regulating cell wall properties. The accumulation of callose under stress or during differentiation acts as an important barrier or resistance mechanism [21,22].

Monocotyledons (monocots) represent a monophyletic clade and one of the major groups among the angiosperms, derived from aquatic and wetland ancestors, e.g., [23,24]. Monocots share distinct primary cell wall chemistry and mechanics, characterized by polysaccharides and proteins that differ significantly from those of eudicots, such as a markedly reduced pectin content. This difference may be associated with increased levels of xyloglucans and recycling of homogalacturonans, reflecting their evolutionary traits and rapid annual growth; see Ref. [25]. The primary cell wall of dicots and non-graminaceous monocots is believed to consist of a cellulose/xyloglucan network that is embedded in and interacting with a pectin network [26]. Furthermore, the pollen cell wall of monocots and aperture configurations are highly consistent with their evolutionary pattern (monosulcate or monosulcate-derived) and distinguish them from the eudicot clade [27,28]. The cell wall plays a crucial role in early embryological processes in pollen, such as microsporogenesis [29]. A phenomenon characteristic of most monocotyledons is successive microsporogenesis, where dyad stage is observed, consisting of two daughter cells separated by a callose wall following the first meiotic division. This process further highlights the distinctiveness of this group compared to eudicots [28,29]. In conclusion, the cell wall structure of monocotyledonous plants presents a compelling area for further study.

Arabinogalactans (AGPs) are a family of hydroxyproline-rich glycoproteins (HRGPs) that serve as molecular markers of gametophytic cells, including the generative and sperm cells, due to their specific spatial distribution during pollen development, especially for their identification and tracking during double fertilization process [30,31,32,33]. Many AGPs function as signalling molecules [34,35,36] and are produced during various stages of pollen and sperm cell development in flowering plants [37,38,39,40,41] and gymnosperms [42,43]. Numerous studies have demonstrated the key role of AGPs in pollen grain germination and pollen tube growth [39,40,42,44,45,46,47]. For instance, AGP6 and AGP11 are crucial for pollen development, while AGP23 and AGP40 are involved in the endosomal machinery of pollen tubes; see Ref. [48].

The set of monoclonal antibodies (JIM8 and JIM13) was chosen based on their known reactivity with distinct AGP epitopes and their earlier successful application in studies on reproductive tissues of angiosperms. However, apart from *Arabidopsis thaliana*, this labeling of AGPs in generative and sperm cells within pollen grains has been reported in only a few dicotyledonous species [49]. Therefore, we decided to include LM2, JIM14, LM14 and MAC207 antibodies—targeting alternative arabinogalactan epitopes—in our analyses and to compare their distribution patterns in the pollen of three selected monocotyledonous species. These antibodies were selected based on their previously described specificity for distinct AGP epitopes and their reported use in reproductive tissues, allowing us to explore possible structural or functional differences in AGP distribution. Unfortunately, no similar studies are available for monocotyledons. Here, to address this gap, the three species *Gagea lutea*, *Galanthus nivalis* and *Ornithogalum nutans* were selected as models. The first species belongs to the Liliaceae family, second species belongs to Amarylidaceae, and *O. nutans*, representing the third taxon, belongs to the Asparagaceae family. To demonstrate AGP pattern in mature pollen grains of monocots, these species were chosen due to their phylogenetic diversity, variations in the size and shape of their generative cells and the existing knowledge of their reproductive processes. Previous embryological and cytological studies of these species have focused on flower development [50], ovule development [51,52,53], fertilization [54] and seed development [55,56,57,58,59]. Pollen meiosis and development of pollen grains have also been extensively studied in these species or close relatives [16,58,59,60,61]. Therefore, this research focuses on mature pollen grains.

The primary aim of this study is to determine whether arabinogalactans are common and widespread markers for the male germ unit or generative/sperm cells in pollen grains of other groups of flowering plants, particularly within the monocotyledon clade. A secondary aim is to identify whether arabinogalactans other than those commonly studied (e.g., those localized with JIM13, JIM8, or LM2 antibodies and others) can serve as markers of generative cells or the male germ unit in monocotyledons. In addition to identifying molecular markers, we were also interested in the spatial relationship between the generative cell and its surrounding vegetative cell, particularly at the membrane. Over the past five decades, the membrane that encases the generative cell and subsequently the sperm cells has been referred to by various names, resulting in inconsistencies and confusion within the literature. Sugi et al. [16] propose the term peri-germ cell membrane (PGCM) to establish uniformity in nomenclature and to enhance future research regarding its functions in pollen development and fertilization. The PGCM is likely to be critical in preserving the integrity of the male germ unit, facilitating communication between vegetative and germ cells and ensuring successful fertilization [16].

Identifying AGPs as reliable markers of the mature male gametophyte in monocots is important for understanding the molecular identity, differentiation and spatial organization of male reproductive cells in this large and diverse clade. Such markers may be useful in studies on pollen development, interspecific comparisons, or the response of reproductive structures to environmental stresses [62,63,64]. Therefore, the central aim of this study was to investigate whether arabinogalactan proteins can serve as molecular markers for the generative cell in monocot pollen grains, as has been proposed earlier for certain dicot species. We hypothesized that specific AGP epitopes, previously described in dicot pollen grains, may also be conserved in monocotyledons and show consistent spatial localization.

## 2. Materials and Methods

### 2.1. Plant Material

Flowers at anthesis of *G. lutea* (L.) Ker Gawl. and *O. nutans* L. were collected from selected sites of Steffens Park—a park near the city center in Gdansk, in the period from March to May 2023. Flowers of *G. nivalis* L. were collected from private collection of M.N. in March 2024. Anthers containing mature pollen grains of all selected species were dessicted in silicon gel (1019691000, Supelco, Merck KGaA, Darmstadt, Germany) up to 48 h and stored in −20 °C. Hydration of the pollen grains took place in a humid chamber at room temperature overnight. The hydrated pollen grains were then placed for 1 h in liquid medium, in order to restore their full germination capacity. Pollen grains of *G. lutea* were for 1 h carried out on medium according to Read [65], supplemented with an appropriate sugar concentration (20% sucrose), in a humid chamber at room temperature. Pollen grains of *O. nutans* and *G. nivalis* were placed for 30 min in BK medium [66] supplemented with 15% sucrose for full restoration.

### 2.2. Histological and Immunochemical Analysis

After activation of pollen grains in liquid medium they were immediately fixed in 1.6% (*w*/*v*) paraformaldehyde (PFA, Sigma-Aldrich, Sigma-Aldrich Sp. z o.o. Poznań, Poland) mixed with 2% (*v*/*v*) glutaraldehyde (GA, Sigma-Aldrich, Sigma-Aldrich Sp. z o.o. Poznań, Poland) in a PIPES buffer overnight at 4° C with an adequate sugar concentration for each analyzed species [67]. The PIPES buffer contained 50 mM PIPES (piperazine-N,N′-bis [2-ethanesulfonic acid], Sigma-Aldrich, Sigma-Aldrich Sp. z o.o. Poznań, Poland), 10 mM EGTA (ethylene glycol-bis[β-aminoethyl ether]N,N,N′,N′-tetraacetic acid, Sigma-Aldrich, Poznań, Poland) and 1 mM MgCl_2_ (Sigma-Aldrich, Sigma-Aldrich Sp. z o.o., Poznań, Poland), pH 6.8. For better fixation of plasmalemmas, pollen grains were was postfixed (0.5% buffered OsO_4_ (Sigma-Aldrich) for 15 min). For analysis of the occurrence of AGPs, the plant material was dehydrated with acetone and embedded in LR White Acrylic Resin (Merck Life Science Sp.z.o.o., an affiliate of Merck KGaA, Darmstadt, Germany). Semithin sections (0.3–0.5 µm thick) were cut on a Leica ultracut UCT ultramicrotome. The rehydrated sections in PBS buffer were blocked with 1% bovine serum albumin (BSA, Sigma-Aldrich) in a PBS buffer and incubated with the following primary antibodies overnight at 4 °C: anti-AGPs [68,69,70,71,72]. All of the primary antibodies were used in a 1:20 dilution. They were purchased from Plant Probes, UK, and the goat anti-rat secondary or anti-mouse secondary antibody conjugated with FITC was purchased from Abcam (Cambridge, UK). The samples were then cover-slipped using a Mowiol mounting medium: a mixture of Mowiol^®^ 4-88 (Sigma-Aldrich) and glycerol for fluorescence microscopy (Merck, Warsaw, Poland) with the addition of 2.5% DABCO (Carl Roth GmbH + Co. KG, Karlsruhe, Germany). They were viewed using a Leica STELLARIS 5 WLL confocal microscope with Lightning deconvolution and Leica DM6000B. Over 50 pollen grains on sections from each species were analyzed for each antibody used twice.

Semithin sections were also prepared for light microscopy (LM). For callose staining, sections were placed in 0.05% decolorized aniline blue (DAB, Merck Life Science Sp.z.o.o., an affiliate of Merck KGaA, Darmstadt, Germany) in PBS buffer for 15 min [73], modified. The cell wall components such as cellulose, and the hemicellulose xyloglucan were labeled using Carbotrace 680 (Ebba Biotech AB, Nobels väg 16 S-171 65 Solna, Sweden; https://www.ebbabiotech.com/products/carbotrace-680?variant=47885141180748, accessed on 7 May 2025). Crystalline cellulose was also labeled using Calcofluor White Stain (Merck Life Science Sp.z.o.o., an affiliate of Merck KGaA, Darmstadt, Germany) for 10 min [72] counterstained with 1 mg/mL in phosphate-buffer saline propidium iodide (PI, Sigma-Aldrich, [74,75]) and visualized with Leica DM6000B DAPI and Rhodamine filter. Photos were acquired as Z stacks and deconvolved using 3 iterations of a 3D nonblind algorithm (AutoQuant™, Media Cybernetics Inc., Rockville, MD, USA). In order to maximize the spatial resolution, the images are presented as maximum projections.

### 2.3. Transmission Electron Microscopy

The pollen grains of *G. lutea* were also examined using electron microscopy, as follows: Pollen grains were fixed in 2.5% glutaraldehyde with 2.5% in a 0.05 M cacodylate buffer (pH 7.0; Sigma-Aldrich Sp. z o.o., Poznań, Poland) for 2 h at room temperature. After fixation, the samples were washed in the same buffer with four changes (15 min each) and postfixed in buffered 1% OsO_4_ at RT for 1h. After rinsing in distilled water, the samples were dehydrated in a graded acetone series and embedded in Epon resin [76]. Ultrathin sections were cut on a Leica ultracut UCT ultramicrotome, stained with UranyLess and lead citrate. Immunogold reactions were conducted utilizing the aforementioned primary antibodies at a dilution of 1:10, incubated overnight at 4 °C. Visualization was achieved with secondary antibodies conjugated to 10 nm colloidal gold particles (1:50, Sigma-Aldrich, Poznań, Poland) for a duration of 3 h at room temperature. The sections were subsequently analyzed using a Fei Tecnai Spirit BioTWIN (Thermo Fisher Scientific, Warszawa, Poland) transmission electron microscope, located at the Bioimaging Laboratory within the Faculty of Biology at the University of Gdańsk. Negative controls for all immunolabelings were created by omitting the primary antibody step, which caused no fluorescence signal in any of the control frames for any stained slides.

## 3. Results

**Figure 1:**
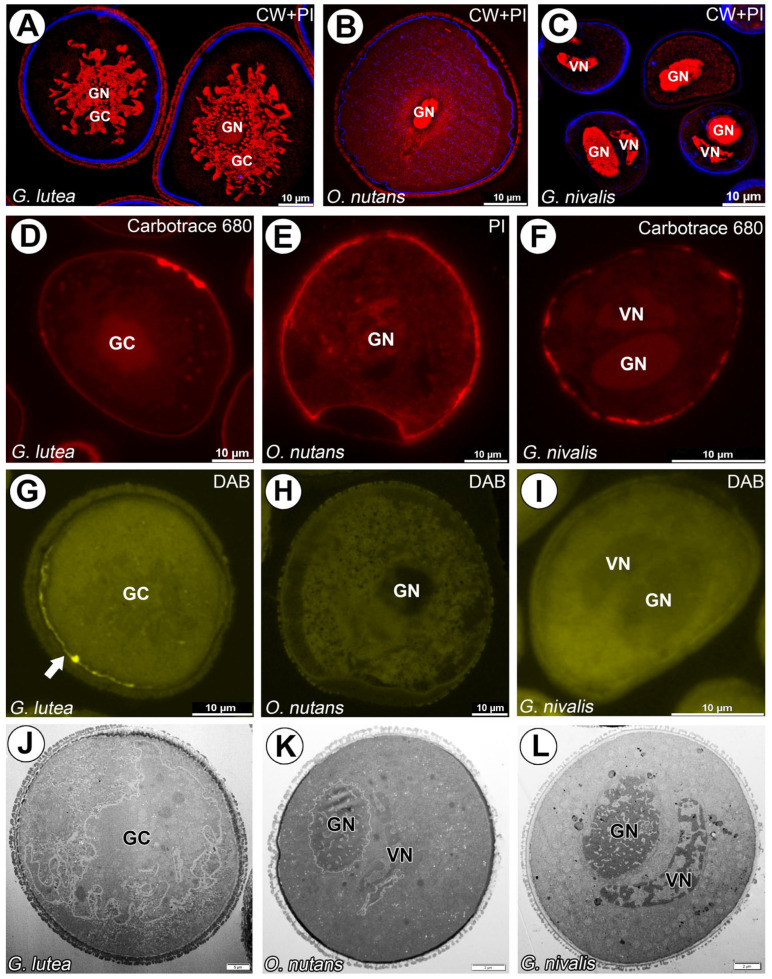
Organization of *G. lutea*, *G. nivalis* and *O. nutans* mature pollen grains. (**A**) A semithin section of the mature pollen grains of *G. lutea*; visible cellulosic intine (blue fluorescence) and folded generative cells (GC) with generative nucleus (GN) stained with propidium iodide (red fluorescence). (**B**) A semithin section of the mature pollen grains of *G. nivalis*; visible cellulosic intine (blue fluorescence) and generative cells with oval generative nuclei (GN) and smaller vegetative nuclei (VN) stained with propidium iodide (red fluorescence). (**C**) A semithin section of the mature pollen grains of *O. nutans*; visible cellulosic intine (blue fluorescence) and a fragment of generative cell with generative nucleus (GN) stained with propidium iodide (red fluorescence). (**D**) A semithin section of the mature pollen grain of *G. lutea*; visible cellulosic intine (red fluorescence) stained Carbotrace 680. (**E**) A semithin section of the mature pollen grain of *O. nutans*; visible cellulosic intine (red fluorescence) stained propidium iodide. (**F**) A semithin section of the mature pollen grains of *G. nivalis*; visible cellulosic intine (red fluorescence) stained Carbotrace 680. (**G**) A semithin section of the mature pollen grain of *G. lutea*; visible only faint signal under intine in particular space (arrow) of callose stained with DAB. (**H**) A semithin section of the mature pollen grain of *O. nutans*; no signal of callose detected within whole pollen grain. (**I**) A semithin section of the mature pollen grain of *G. nivalis*; no signal of callose detected within whole pollen. (**J**) Ultrastructure of mature pollen grain of *G. lutea*; (GC) generative cell with visible submerged and separated from vegetative cell cytoplasm. (**K**) Ultrastructure of mature pollen grain of *O. nutans* with visible (GN) generative and vegetative (VN) nuclei. (**L**) Ultrastructure of mature pollen grain of *G. nivalis* with visible (GN) generative and vegetative (VN) nuclei.

The generative cell of *G. lutea* found in the mature pollen grain has a distinctly undulating surface. The lobed cell nucleus, usually containing a single, large nucleus, occupies a central position and its interior is filled with diffuse chromatin. It is immersed in the vegetative cell and separated from its cytoplasm by the PGCM (Figure 1A,D,G,J). The generative cell of *O. nutans* is significantly elongated with curved ends and contains relatively small nucleus in the center (Figure 1B,E,H,K). Mature pollen grain of *G. nivalis* contains large, lens shaped generative cell with folded ends and centrally located oval nucleus (Figure 1C,F,I,L).

In all studied species no cellulosic component surrounding generative cells was observed using Calcofluor White (Figure 1A–C) and Carbotrace 680 staining (Figure 1D,F). Decolorized aniline blue (DAB) staining showed lack of callose in all analyzed; in some pollen grains, only a faint signal was located partially under the sporoderm (Figure 1G–I).

**Figure 2:**
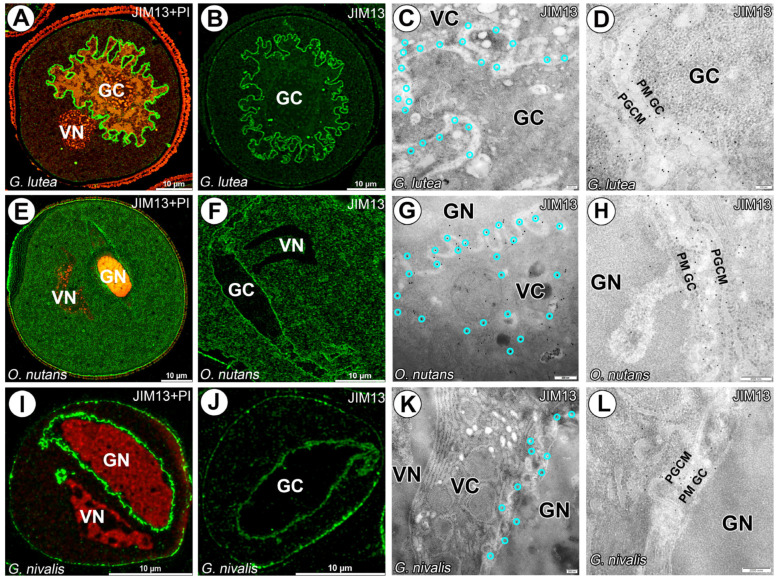
Arabinogalactan proteins detected with JIM13 mAb in *G. lutea*, *G. nivalis* and *O. nutans* mature pollen grains. (**A**–**D**) Arabinogalactan proteins detected surrounding *G. lutea* generative cell and within whole cytoplasm of vegetative cell. (**E**–**H**) Arabinogalactan proteins detected surrounding *O. nutans* generative cell and within whole cytoplasm of vegetative cell. (**I**–**L**) Arabinogalactan proteins detected surrounding *G. nivalis* generative cell. and within whole cytoplasm of vegetative cell. (GC)—generative cell, (GN)—generative nucleus, (VC)—vegetative cell nucleus, (PGCM)—peri-germ cell membrane, (PM GC)—plasma membrane of generative cell. (**A,E**,**I**) Pollen grains were counterstained with propidium iodide (red fluorescence) to localize (GN) and (VN). Representative gold grains were marked with a light cyan circle for better clarity.

**Figure 3:**
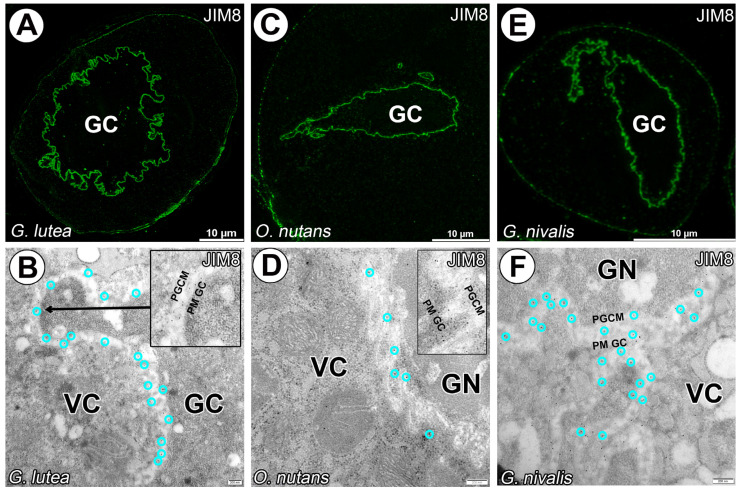
Arabinogalactan proteins detected with JIM8 mAbs in *G. lutea* (**A**,**D**), *O. nutans* (**B**,**E**) and *G. nivalis* (**C**,**F**) mature pollen grains. (**A**) Arabinogalactan proteins recognized with JIM8 mAb surrounding only *G. lutea* generative cell. (**B**) Arabinogalactan proteins recognized with JIM8 mAb surrounding only *O. nutans* generative cell. (**C**) Arabinogalactan proteins recognized with JIM8 mAb surrounding only *G. nivalis* generative cell. (**D**) Arabinan epitope recognized with JIM8 mAb surrounding only *G. lutea* generative cell, the zoomed-in area presented in (**D**) is taken from different regions of the same pollen grain (**E**) Arabinan epitope recognized with JIM8 mAb surrounding only *O. nutans* generative cell. (**F**) Arabinan epitope recognized with JIM8 mAb surrounding only *G. nivalis* generative cell. (GC)—generative cell, (GN)—generative nucleus, (VC)—vegetative cell nucleus, (PGCM)—peri-germ cell membrane, (PM GC)—plasma membrane of generative cell. Representative gold grains were marked with a light cyan circle for better clarity.

The AGPs epitopes recognized by the JIM13 and JIM18 mAbs were detected in surrounding generative cells for all studied species. No distinct labeling was observed for vegetative nuclei. Signal between both plasmalemmas of vegetative and generative cells was distinct and homogeneous (Figure 2 and Figure 3). Only for JIM13 labeling (against β-GlcA-(1-3)-α-GalA-(1-2)-Rha epitope) signal was observed in cytoplasm of vegetative cell cytoplasm, particularly for *O. nutans* (Figure 2E–H).

**Figure 4:**
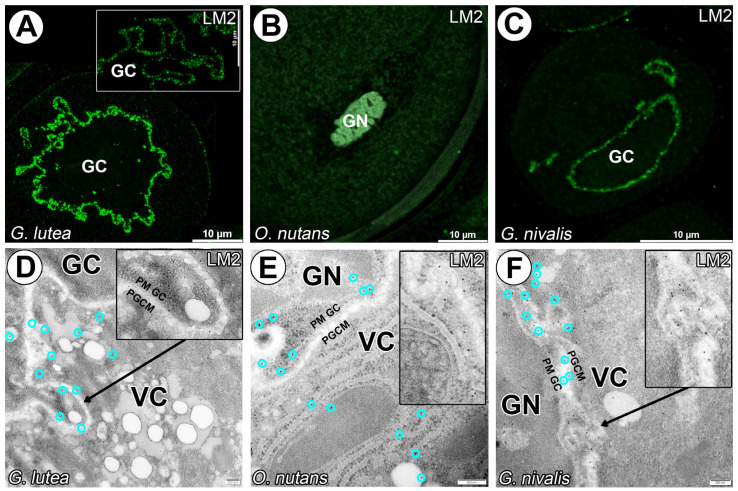
Arabinogalactan proteins detected with LM2 mAb in *G. lutea*, *G. nivalis* and *O. nutans* mature pollen grains. (**A**,**D**) Arabinogalactan proteins detected surrounding only *G. lutea* generative cell (GC). ((**B**,**E**), the zoomed-in area presented in (**E**) is taken from different regions of the same pollen grain). Lack of signal recognized with LM2 mAb in generative cell and vegetative cell cytoplasm (green fluorescence), gray signal represents autofluorescence of generative cell nucleus (GN) and sporoderm. *O. nutans* generative cell. (**C**,**F**) Arabinogalactan proteins detected surrounding only *G. nivalis* generative cell (GC). (PGCM)—peri-germ cell membrane, (PM GC)—plasma membrane of generative cell. Representative gold grains were marked with a light cyan circle for better clarity.

A carbohydrate epitope containing B-linked glucuronic acid recognized with LM2 mAb occured in *G. lutea* and *G. nivalis* generative cells, but very little in *O. nutans* (Figure 4E). The fluorescence signal of this labeling is more granular than uniform in the space between plasmalemmas of two cells in pollen grains (Figure 4).

**Figure 5:**
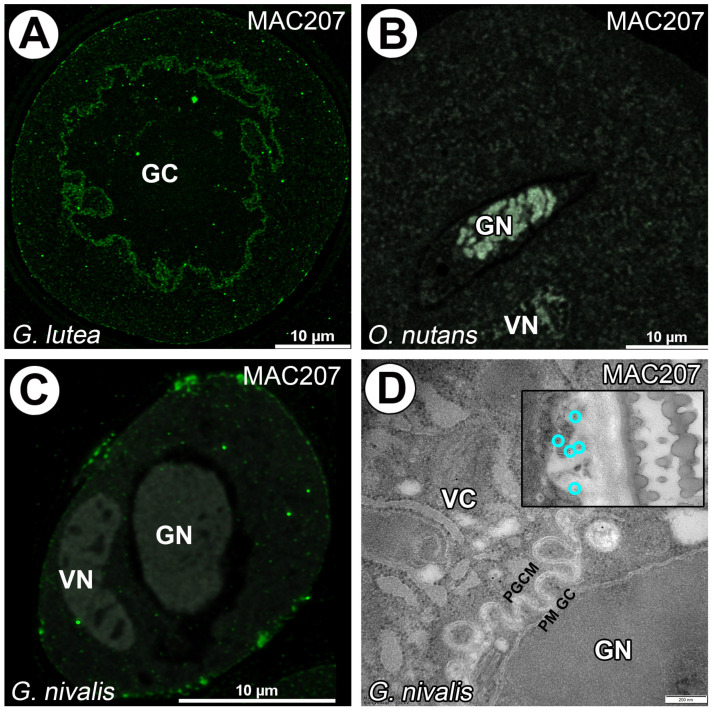
Arabinogalactan proteins detected with MAC207 in *G. lutea* (**A**), *O. nutans* (**B**) and *G. nivalis* (**C**,**D**) mature pollen grains (green fluorescence). (**A**) Arabinogalactan proteins recognized with MAC207 mAb surrounding *G. lutea* generative cell and within of vegetative cell cytoplasm. (**B**) Lack of signal from MAC207 mAb in *O. nutans* pollen grain. (**C**) Lack of signal from MAC207 mAb surrounding *G. nivalis* generative cell; vegetative cell cytoplasm only has visible patches of signal under sporoderm (**D**). (GC)—generative cell, (GN)—generative cell nucleus, (VN)—vegetative cell nucleus. (**B**,**C**)—gray signal represents autofluorescence of generative and vegetative cell nucleus and sporoderm. (PGCM)—peri-germ cell membrane, (PM GC)—plasma membrane of generative cell. Representative gold grains were marked with a light cyan circle for better clarity.

For *O. nutans* and *G. nivalis* mature pollen grains epitope β-GlcA1->3-α-GalA1->2Rha recognized with MAC207 mAb was not observed in surrounding generative cells and in the vegetative cell cytoplasm in contrast to generative cell of *G. lutea* were signal was detected. Some particularly clusters of signals were also located under sporoderm (Figure 5A–D).

**Figure 6:**
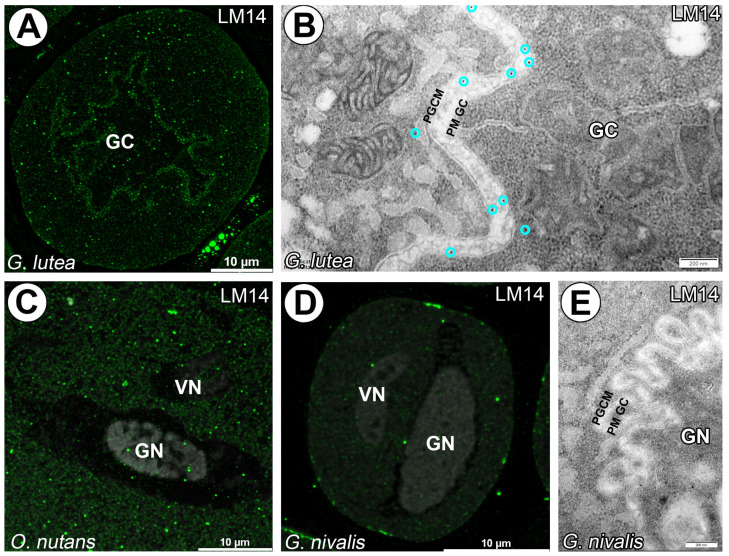
Arabinogalactan proteins recognized with LM14 mAb surrounding *G. lutea* generative cell and within of vegetative cell cytoplasm (green fluorescence). (**A**,**B**) Lack of signal from LM14 mAb in *O. nutans* generative cell, punctate signal only detected in vegetative cell cytoplasm. (**C**) Lack of signal from LM14 mAb surrounding *G. nivalis* generative cell, vegetative cell cytoplasm, only visible patches of signal under sporoderm (**D**,**E**). (GC)—generative cell, (GN)—generative cell nucleus, (VN)—vegetative cell nucleus, (PGCM)—peri-germ cell membrane, (PM GC)—plasma membrane of generative cell. (**C**,**D**)—gray signal represents autofluorescence of generative and vegetative cell nucleus and sporoderm. Representative gold grains were marked with a light cyan circle for better clarity.

Similar observations were made utilizing LM14 labeling for carbohydrate epitope containing Β-linked glucuronic acid recognition. Here, granular signal was observed only for *G. lutea* generative cell and in cytoplasm of *G. lutea* and *O. nutans* pollen grains (Figure 6A–C). Mature pollen grains of *G. nivalis* did not have significant signal detected (Figure 6D,E).

**Figure 7:**
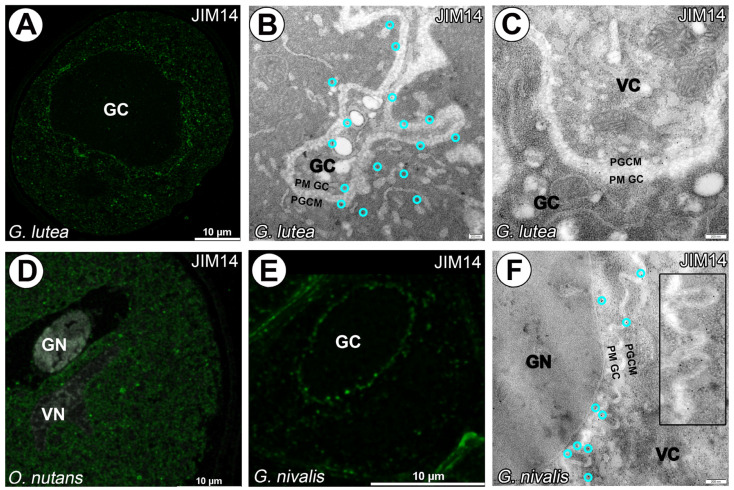
Arabinogalactan proteins recognized with JIM14 mAb surrounding *G. lutea* generative cell and within of vegetative cell cytoplasm (green fluorescence). (**A**–**C**) Lack of signal from JIM14 mAb in *O. nutans* generative cell, punctate signal only detected in vegetative cell cytoplasm. (**D**) Arabinogalactan proteins recognized with JIM14 mAb only surrounding *G. nivalis* generative cell (**E**,**F**), the zoomed-in area presented in (**F**) is taken from different regions of the same pollen grain). (GC)—generative cell, (GN)—generative cell nucleus, (VC)—vegetative cell, (VN)—vegetative cell nucleus, (PGCM)—peri-germ cell membrane, (PM GC)—plasma membrane of generative cell. (**D**)—gray signal represents autofluorescence of generative and vegetative cell nucleus and sporoderm. Representative gold grains were marked with a light cyan circle for better clarity.

Arabinogalactan proteins identified by the JIM14 monoclonal antibody are primarily localized around the generative cell of *G. lutea* and within the cytoplasm of the vegetative cell (Figure 7A–C). In contrast, a lack of signal from the JIM14 monoclonal antibody was noted in the generative cell of *O. nutans*, with only a punctate signal detected in the cytoplasm of the vegetative cell (Figure 7D). Furthermore, arabinogalactan proteins recognized by the JIM14 monoclonal antibody were solely visualized surrounding the generative cell of *G. nivalis* (Figure 7E,F).

**Figure 8:**
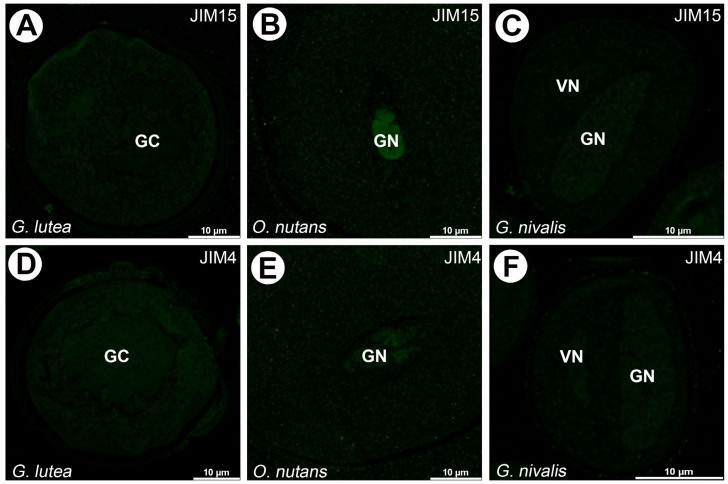
Absence of signal of JIM15 (**A**–**C**) and JIM4 (**D**–**F**) observed in all examined species. (GC)—generative cell, (GN)—generative nucleus, (VC)—vegetative cell nucleus.

No signal from JIM4 and JIM15 mAbs was observed in all studied pollen grains species (Figure 8).

**Figure 9:**
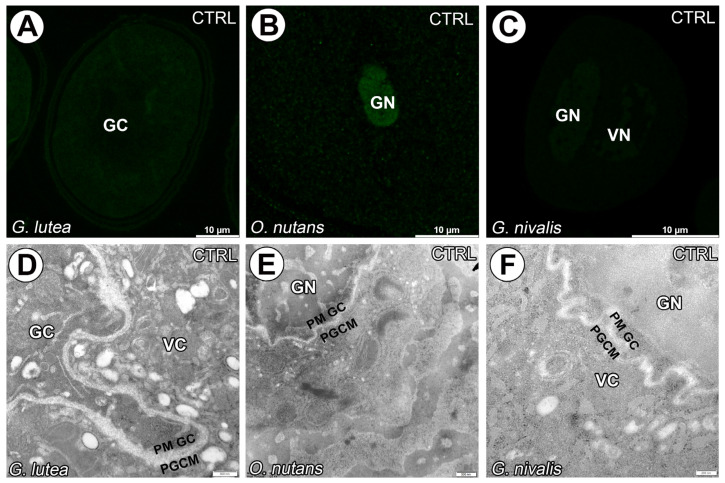
Negative control reaction of immunofluorescence and immunogold experiments omitting primary antibody. Lack of specific signal in all studied species (**A**–**F**). (GC)—generative cell, (GN)—generative nucleus, (VC)—vegetative cell nucleus, (PGCM)—peri-germ cell membrane, (PM GC)—plasma membrane of generative cell.

The negative control reaction of immunogold experiments, which involved the omission of the primary antibody, demonstrated a lack of specific signal across all studied species (Figure 9).

## 4. Discussion

### 4.1. Cell–Cell Junction or Cell Wall Surrounding Generative Cell?

During the analysis of AGP localization, we noticed that the signal often appears at the contact site between the generative cell membrane and the cytoplasm of the vegetative cell. This observation led us to take a closer look at how these two membranes are organized and whether this interface could play a role in cell-to-cell communication. It is believed, that in mature pollen grains, the space between the generative and vegetative cells is a cell–cell junction and their membranes are parallel [16,77,78]. Structural and chemical nature of this connection has been a topic of considerable debate. However, subsequent TEM investigations increasingly supported the presence of a proper cell wall see [79]. Lancelle and Hepler [80] for instance observed patches in the space between the apoplast between the PGCM and the GC plasma membrane in *Lilium longiflorum* pollen tubes, interpreting them as plasmodesmata. Nevertheless, the composition of these structures was not identified. The hypothesis of a cell wall was further supported by the juxtaposition of two membranes and the presence of matrix material [81]. The results of rapid freezing and freeze-substitution techniques have provided new insights into the nature of this space. These approaches have demonstrated that the separation comprises two membranes, which appear straighter and more clearly separated in pollen grains and pollen tubes compared to the uneven and irregular profiles observed after aldehyde fixation [82,83,84]. Observations of isolated generative cells also suggest that the extracellular matrix surrounding them is incomplete [83]. In the present study, we observed spaces between membranes in mature pollen grains of *G. lutea*, *G. nivalis* and *O. nutans*. The spaces between the apoplast between the PGCM and the GC plasma membrane exhibited uniform or irregular structures, conceivably due possibly due to chemical fixation. This observation indicates that chemical fixation can alter the preservation of cell wall structures and plant secretions, particularly when compared to material preserved by high-pressure freezing [85,86,87,88].

### 4.2. Presence of Β-Glucans in Generative Cell

Following pollen mitosis I (PMI), when the generative cell initially remains appressed to the sporoderm, it is transiently surrounded by callose. During detachment, this deposited component dissolves, allowing the generative cell to become fully immersed in the vegetative cytoplasm [77,81,89]. However, Heslop-Harrison [90] and Tanaka [91] reported the presence of callose in generative and sperm cells of *Dactylorchis fuchsii* (Druce) Soó and *D. purpurella* (T.Stephenson & T.A.Stephenson) Verm., and, respectively, *Lilium longiflorum* Thunb. Studies on an uncharacterized pollen-specific SKS-like protein (PSP231) in cotton (*Gossypium hirsutum* L.), revealed that specifically during the post-pollen mitosis stage, activation of callose biosynthesis is crucial for promoting pollen maturation. However, in mature pollen grains only weak staining of callose was detected, pointing to the germination pore [92]. Our findings are consistent with the view that the generative cells in mature pollen grains are devoid of callose. A faint signal observed just beneath sporoderm may indicate the future site of pollen tube emergence. Overall, the generative cell exhibits variability in size and shape within species and can be enclosed by various types of thin pectocellulosic cell walls [93]. Cresti et al. [81] observed electron-dense material surrounding the generative cell in mature pollen grains of *Euphorbia dulcis* L., suggesting the presence of a cell wall. However, this structure was described as neither typical nor solid and pectocellulosic. In the studied species, we did not observe cellulosic material surrounding the generative cells of the studied species using calcofluor and Carbotrace staining. These results suggest that, in the species studied, the generative cells are not surrounded by any cell wall. The PM of the generative cell (PM GC) membrane appears to be closely associated with the membrane of the vegetative cell (PGCM).

### 4.3. AGPs in Generative Cell of Angiosperms

To date, only two studies have reported the presence of arabinogalactan proteins in the pollen of monocotyledonous species, to which *G. lutea*, *G. nivalis* and *O. nutans* belong. The first study conducted by Southworth and Kwiatkowski [40] examined the presence of AGPs at the cell surface of *Lilium* sperms and generative cells. Isolated generative and sperm cells from pollen tubes of *Lilium longiflorum* showed surface patches stained with JIM8 and JIM13 mAbs. Nuclei and other cytoplasmic components of generative and sperm cells were devoid of any signal. It should be emphasized that such isolated cells may have had remnants of membranes and cytoplasm of the vegetative cell on their surface, so such studies require comparison with non-isolated cells. In the present study on selected monocots, similar findings were observed, however not on isolated generative cells. The labeled AGPs surrounding the generative cells showed a strong, non-speckled signal in the studied species. These results confirm that the pattern of AGPs labeled with the aforementioned antibodies appears very similar to many angiosperms and provides a reliable marker for generative and sperm cells among them. The second study, conducted by Wisniewska and Majewska-Sawka [94] investigated AGPs in *Lolium perenne* L. (from the Poaceae family) anthers and pistils. In anthers, microspores at the uninucleate stage were observed but not mature pollen grains. During this developmental stage, JIM13 mAb was localized within the entire microspore cytoplasm, no signal from the nuclei of microspore was reported. As young microspores of angiosperms are released from the tetrads, JIM13 mAb signal is detected in primexine and on the outer surface, where the intine wall will later form [95,96,97,98,99,100].

In three species studied AGPs epitopes localized with JIM8 and JIM13 mAbs gave strong homogeneous signal, suggesting that the apoplast between the PGCM and the GC plasma membrane is filled with carbohydrate and glycan residues. Similar results were obtained for the species previously mentioned in this article. Differences in the detected signals of AGPs in the studied material, localized using LM2, JIM14, LM14 and MAC207 mAbs can indicate interspecific variations.

Nakamura and Miki-Hirosige [101] postulated that in the pollen grain of *Lilium longiflorum*, the apoplast between the PGCM and the GC plasma membrane is formed with polysaccharide vesicles derived from lipid bodies, coated vesicles and numerous rough ER which may reflect ER involvement in cell wall formation. JIM 13 and LM2 mAbs employed for labeling both Golgi vesicles and vesicles in the paramural body, indicates the involvement of these vesicles in pollen tube wall formation [41]. These observations support the view that in various species, the substance of the apoplast between the PGCM and the GC plasma membrane is contributed by different sets of organelles. In *Tradescantia paludosa* E.S. Anderson & Woodson, vesicles containing pectins, together with the forming phragmoplast, assemble the apoplast between the PGCM and the GC plasma membrane enclosing the generative cell [102,103]. Overall, the presence of arabinogalactans and arabinan residues surrounding generative cell are coincides with earlier observations of polysaccharides revealed by PAS staining [104]. These findings highlight the role of AGPs not only as evolutionarily conserved structural components, but also as molecular markers that allow identification of generative cells across angiosperms. While our results provide novel insights into AGP localization in mature monocot pollen, we acknowledge several limitations. First, this study was restricted to three species and selected epitopes, which may not represent the full diversity of AGP distribution across monocots. Second, our analysis focused exclusively on mature pollen grains, omitting developmental stages that could offer a more dynamic view of AGP biosynthesis and transport. Our future work will include a broader taxonomic sampling and developmental analyses to better address the function of AGPs during male gametophyte differentiation. Mature pollen grains are also sensitive to environmental stresses, which can directly influence pollen viability and pollen tube growth [64]. Therefore, identifying reliable molecular markers associated with structure and cell-to-cell communication within the male germ unit may be valuable not only for basic research but also for applied studies.

### 4.4. Evolutionary Perspective of AGPs in Generative Cell

Our research complements existing studies on the presence of arabinogalactans in generative cells in alternative clades of eudicots and extends findings previously discussed for monocotyledonous plants. Immunochemical analyses of the pollen grains and pollen tube walls, in case of AGPs, remain largely absent in gnetophytes [105]. Studies addressing angiosperms in generative or sperm cells distant phylogenetic clades, such as gymnosperms and lower vascular plants, are scarce but provide valuable insights. Nevertheless, in the pollen tubes of the conifer *Pinus densiflora* S. et Z., the generative cell was abundantly labeled with LM2 mAb but no signal was detected with JIM13 mAb. Both AGP epitopes recognized by these mAbs were detected in pollen intine [41]. Similarly, in *Pinus bungeana* Zucc. ex Endl. and *Picea wilsonnii* Mast. intine were also labeled with LM2 mAb [106]. Arabinogalactan proteins localized with JIM13 and LM2 mAbs were observed only in intine and the pollen tube wall of the 14 gymnosperms examined, and a mixed β-glucan was abundantly present in *Ginkgo biloba* L. [43]. In a subsequent study presented by Rafińska et al. [107] on the mature, hydrated pollen grain of *Larix decidua* Mill., AGPs (epitopes recognized with JIM4, JIM8, JIM13 and LM2 mAbs) were present in the cytoplasm of pollen cells, the signal for JIM4 mAb was the weakest. It is therefore clear that the AGP localization in mature pollen grains of gymnosperms varies considerably between gymnosperms themselves but overall corresponds to flowering plant patterns. Angiosperms and gymnosperms both utilize pollen carrying sperms/sperm cells to facilitate fertilization. For gymnosperms, AGPs in pollen that contain glucuronic acid and a high level of uronic acids may also participate in pollen growth [42]. Additional studies on the early angiosperm *Trithuria submersa* Hook.f. (Hydatellaceae) have added to the knowledge of the presence of AGPs in anthers, where intense labeling obtained with JIM8 and JIM13, mAbs was located in the intine wall of this species at the aperture, again pointing future pollen tube emergence site [35]. In lower vascular plants, AGPs associated with male reproductive structures have been reported during sperm maturation in the fern *Ceratopteris richardii* Brongn. [108,109,110]. The localization of AGPs recognized with JIM8 and JIM13 mAbs on the plasmalemma and in the extraprotoplasmic matrix of sperm cells in this fern appears similar to the results obtained for generative cells in the studied monocot species in the present work. The dynamic arabinogalactan populations in spermatid walls fluctuate during differentiation and were associated with high concentrations of arabinose in *Physcomitrium patens* (Hedw.) Mitt. [111,112] and *Aulacomnium palustre* (Hedw.) Schwägr. [110]. The studies suggest that in these species the pool of AGPs may vary across generations and may be crucial for flagella formation in spermatids of ferns. Taken together, AGPs are a widespread and evolutionarily ancient class of glycoproteins, playing a significant role in key adaptations for reproduction in most land plants. This evolutionary conservation, together with their localization between gametophytic cells, supports the interpretation of AGPs as molecular markers associated with the differentiation of the male gametophyte. Their presence may reflect developmental mechanisms or interactions essential for successful fertilization [63]. Our findings complement existing phylogenetic knowledge by bridging the gap between data reported for gymnosperms and those available for dicotyledonous species, thereby broadening the evolutionary perspective on the role of AGPs in male gametophyte development. Although our results show a clear AGP signal at the boundary between the generative cell and the vegetative cytoplasm, we cannot conclusively determine whether the epitopes are located within the PGCM, the generative cell plasma membrane, or the intervening apoplastic space. Further ultrastructural and biochemical studies will be needed to resolve their precise subcellular localization.

## 5. Conclusions

1. Our work is the first to demonstrate the presence of AGPs between the generative and vegetative cells in pollen of monocotyledonous species. These findings suggest that certain AGPs detected with JIM8 and JIM13 may serve as a marker of the generative cell not only in dicotyledons but also in monocotyledons. Further ultrastructural studies are needed to determine the exact localization of AGPs in the species studied; they are either located in the intercellular matrix or sub-membranously in the generative or vegetative cell.
2. We found differences between LM2, LM14, JIM14 and MAC207 in the studied species. These results may suggest interspecific variations and require further investigations among monocot species.
3. We did not detect any signal with JIM4 and JIM15 antibodies, which may be due to interspecific variations or AGPs not specific to these species or even to the mature pollen grains of monocots.

## Data Availability

The data presented in this study are available on request from the corresponding author.

## Abbreviations

AGPs—arabinogalactan proteins, LM—light microscope, TEM—transmission electron microscopy.
